# An Embedded Sensory System for Worker Safety: Prototype Development and Evaluation

**DOI:** 10.3390/s18041200

**Published:** 2018-04-14

**Authors:** Chunhee Cho, JeeWoong Park

**Affiliations:** Department of Civil and Environmental Engineering and Construction, The University of Nevada, Las Vegas, NV 89154, USA; chunhee.cho@unlv.edu

**Keywords:** construction, construction worker, safety, awareness, communication, sensing

## Abstract

At a construction site, workers mainly rely on two senses, which are sight and sound, in order to perceive their physical surroundings. However, they are often hindered by the nature of most construction sites, which are usually dynamic, loud, and complicated. To overcome these challenges, this research explored a method using an embedded sensory system that might offer construction workers an artificial sensing ability to better perceive their surroundings. This study identified three parameters (i.e., intensity, signal length, and delay between consecutive pulses) needed for tactile-based signals for the construction workers to communicate quickly. We developed a prototype system based on these parameters, conducted experimental studies to quantify and validate the sensitivity of the parameters for quick communication, and analyzed test data to reveal what was added by this method in order to perceive information from the tactile signals. The findings disclosed that the parameters of tactile-based signals and their distinguishable ranges could be perceived in a short amount of time (i.e., a fraction of a second). Further experimentation demonstrated the capability of the identified unit signals combined with a signal mapping technique to effectively deliver simple information to individuals and offer an additional sense of awareness to the surroundings. The findings of this study could serve as a basis for future research in exploring advanced tactile-based messages to overcome challenges in environments for which communication is a struggle.

## 1. Introduction

Safety is one of the most critical challenges in the construction industry. This industry suffers from a high number of accidents. In fact, the number of accidents in construction is the highest among all U.S. industries. In 2015, the number of fatal injuries in the private construction industry was 937, which was the highest since 2008 (973 cases) [1]. This 2015 rate was 4% greater than 2014, which indicates that current ongoing efforts to implement adequate safety measures are inadequate. 

In order to provide proper safety measures, construction researchers have taken into account recent technological advances such as information and sensing technology as well as associated computational techniques. Using vision technology, researchers accomplished accurate real-time detection of workers [2,3,4,5], tracking of equipment and resources [6,7,8], productivity analysis [9], and a construction hazard avoidance system [10]. Wireless signal-based sensors have been explored for solutions towards labor tracking [11,12,13] and safety tracking [14,15,16]. Motion sensors are another type of technology that have been investigated for their capability of accurate measurement of physical motions, which created various applications such as posture recognition [17], tracking [18], and fall hazard detection [19,20,21,22]. Recently, sound technology demonstrated its ability to recognize equipment through machine-leaning-based approaches by relying on its unique sound [23]. Most sensor-based research has focused on developing and validating methods for understanding construction site situations by taking advantage of the capability of sensors to collect data continuously. Research that attempted to address real-time safety issues is also limited in that research has focused on identifying and detecting safety issues on a system level, but has not closed the last link. This link involves delivering the message of detected hazards to workers, which is otherwise known as real-time hazard perception. 

Construction sites are generally very loud, dynamic, complicated, and prone to hazards, which may interrupt workers’ ability to communicate (e.g., perceive hazard information). Harsh environmental conditions in construction sites can blunt workers’ senses, which limits their ability to execute preventative measures for avoiding potential accidents. In the absence of workers perceiving impending hazards, identifying and detecting by using advanced technological methods is meaningless and preventative reactions may not take place in time. The significance of the inability to perceive hazards properly among construction workers is clear. However, current practices and state-of-the-art construction research using technology have not found adequate resolutions to this problem. 

This paper proposes a prototype for a wearable tactile sensory system to address issues resulting from limited sensing abilities of construction workers. The conceptual idea of an embedded tactile sensory system is built on neurological evidence that a human brain can extend a perception range by learning through repeated actions and adding new perception skills through different sensory channels [24,25]. In other words, if patterned stimuli—for example, tactile signals in this study—carry information (e.g., construction hazard types), the brain can analyze them and understand their meanings by using repeated training. The construction industry can take advantage of this finding to improve the level of awareness of construction workers. This study used the following steps:

(1) Developed a customized tactile sensory system for formulating a tactile language system,
(2) Explored signal parameters to identify basic unit signals that could be perceived in a short amount of time (i.e., less than 0.5 s per unit signal) by using three parameters: signal intensity, signal length, and delay between consecutive pulses, and
(3) Analyzed the test data from these two experiments.

## 2. Literature Review

### 2.1. Sensor-Based Research for Construction Safety

Recent research has focused on using various sensing technologies to capture real-time on-site data to offer safety monitoring systems [14,26,27,28,29,30] for workers who are engaged in their activities and may be oblivious to their surroundings and nearby hazards. Therefore, they may have an accident. On a system level, researchers made significant advances in automatically detecting/monitoring safety issues. Despite these advances, however, such developments have not helped individual workers possibly because previous research has not explored methods that could deliver system-level safety information to these workers promptly and effectively.

In fact, a few of the researchers who have developed safety monitoring systems have not completely overlooked the significance of notifying workers about detected potential incidents. A few studies have used alerts [27,31,32] and visualization [33,34] to draw attention to the importance of real-time hazard perception at an individual level. However, because the methodology of these studies have not been verified against the challenges prevalent in construction sites—such as noise, dynamic changes, and engaged workers oblivious to their surroundings—practitioners often question the applicability of such methods because they may not be effective in many situations. Recent research also identified potential issues of safety technologies in delivering alerts to worker [35,36] due to failure in alerting workers in a harsh construction environment. In sum, no research in construction safety has covered methods adequately for providing system-level safety information to individual workers. Therefore, this research aims to develop and validate an embedded sensory system that can improve construction workers’ communication in a harsh environment.

### 2.2. Background for Artificial Sensory Research

Previous neurological research demonstrated that artificially constructed senses can replace a lost sense such as losing sight or hearing [24,37,38]. Blind people can see by obtaining information obtained by eyes in other substitutive forms such as by sound and vibratory signals [39]. Continuous training for seeing based on sound and/or vibratory signals can achieve a successful connection to the brain for communicating and interpreting sound and/or vibratory signals similar to how it is done with functional eyes. An important point is that researchers have investigated developing the substitution of a lost sense. In fact, the brain receives electrochemical signals regardless of what part of the body the signals come from and how the signals are collected. 

Chekhchoukh et al. [40] explained this concept as many peripherals connected to the human brain. This implies that the sensory organs that humans have are only input organs. For the brain, the senses simply provide electrochemical information that the brain needs to receive and interpret in order to perceive the world. In other words, the process from sensing things to brain perception is a result of combinations of interactions between organs and nerves and their information delivery flow. This implies that eyes for vision, ears for sound, and a tongue for taste only partially sense things. When a human senses something, the sensed information is received by receptors and the associated information is converted to electrochemical signals and sent to the brain. 

For people with inborn disabilities or who have lost certain senses such as sight (blindness) or hearing (deafness), the initiating function is left disabled and their bodies are unable to trigger signals through these receptors. Researchers [25,41] have investigated ways of triggering signals that are alternative to lost senses by attempting to mimic what could have been sensed otherwise. In this way, the human body can develop equivalent electrochemical signals, which the brain can perceive as it would do for the lost senses. Many researchers have demonstrated that such alternative signals could function as intended and thus could be used as a substitute for the missing senses.

## 3. Objective and Scope

The primary objective of this research was to investigate a methodology for a prototype embedded (wearable) sensory system that directly communicates with individual construction workers who have limited sensing abilities including both vision and hearing. By directly communicating with workers, this system could offer them additional safety awareness of their surroundings. The major goal of this system was to overcome limited sensing abilities of the workers due to loud and dynamic construction environments. However, prior to investigation on a complete level of communication with construction workers for safety purpose, it is necessary to develop the fundamental understanding of tactile signals. This study attempted to understand the basic parameters of tactile signals (i.e., signal intensity, signal length, and delay between consecutive pulses) with respect to the level of resolution in digitization and perceptional sensitivity. 

The hypothesis of this research was that real-time safety information could be transferred to workers through an embedded sensory system in order to expand the sensing domain of workers beyond vision and sound. To test this hypothesis, this study used the following research steps: (1) the tactile signal was digitized so these digitized signals could be perceived by workers by using an embedded sensory system, (2) communicable information units of digitized tactile signals were identified by creating a tactile-based communication system and by developing various profiles of signals to convey piece wisely digitized information. For improved safety, it was crucial to determine the types of signals that humans could perceive in a short period of time. The experiments of this study measured the perception accuracy of workers with regard to the transmitted signals. The results of these experiments were used to investigate the signal profiles in terms of identified parameters and their ranges that could lead to distinguishable tactile messages for effective communication.

The scope of this research included the development of a prototype tactile communication system, tests of various signals, and measurements of the perception rates of test subjects. The tested parameters for constructing signal profiles were intensity, signal length on each pulse, and delay between pulses. Such an embedded sensory system should generate signals that can be recognized promptly by workers without requiring a significant amount of time to interpret the signals. 

This factor—quick recognition—led to a time-constraint challenge, which was one of the major differences of this study when compared to earlier neurological studies. To overcome the time constraint, the research team adopted a method of mapping. First, a certain set of signals were assigned to certain contexts. Afterward, the signals were indexed and mapped to the corresponding contexts for quick message delivery. The experiments were designed to reveal the perception abilities of the test subjects and their sensitivity levels of perception.

## 4. Method

### 4.1. Body Stimulus and Human Recognition

**Figure 1:**
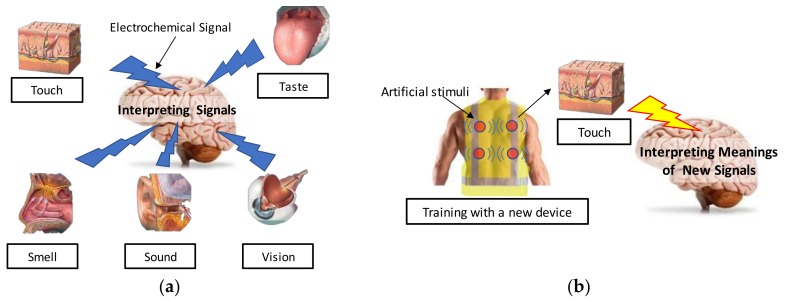
Information processing of a human using (**a**) a common sensing mechanism and (**b**) addition of a new sensory device.

Humans recognize the world mainly through five sensing abilities. This includes taste, sight, touch, smell, and sound. Unfortunately, construction workers mainly rely on two senses, which are sight and sound, to perceive their surroundings in a construction site. These two sense are major sources for them to understand their physical environments while performing their daily work activities, operating equipment, and protecting themselves from hazards. Although these senses are critical for various tasks, they are often hindered because of the nature of construction sites, which are dynamic, loud, and complicated. Perceived information—for example, things that can be tasted, seen, touched, smelled, and heard—are converted to electrochemical signals that flow through the human body and reach the brain. Such information is perceived as electrochemical data by the human brain and the brain immediately starts to interpret the meaning of the transmitted electrochemical data, which is shown in Figure 1a. As shown by previous neurological studies [37,38], humans can develop senses in different channels by training the brain and being triggered by certain stimuli as well as understanding the corresponding information paired with stimuli, which is shown in Figure 1b. 

This study used an approach similar to the neurological studies mentioned previously [37,38]. However, it deviated from the intention to fully develop new senses as a substitution for the lost or missing senses. Full development of a new sense to compensate for a lost sense requires a method of substitution to deliver messages at a comprehensive level. This typically requires time for interpretation. However, when safety in the construction site is imperative, full delivery of messages may not be ideal. 

Because of the time constraints imposed by a construction site, this study explored vibratory signals at a fundamental level in order to understand perception ability. This study involved the following four factors.

(1) We developed a prototype tactile-based communication system,
(2) Developed artificial tactile stimuli by creating various vibratory signals and refining such parameters as intensity, signal length, and delay between consecutive pulses profiles,
(3) Extensively tested the signals that were created and their parameters to identify easily distinguishable profiles and their characteristics, and
(4) Used the identified tactile signals to further test the ability to directly inform the test subjects of an impending hazard.

### 4.2. Apparatus: System Components

**Figure 2:**
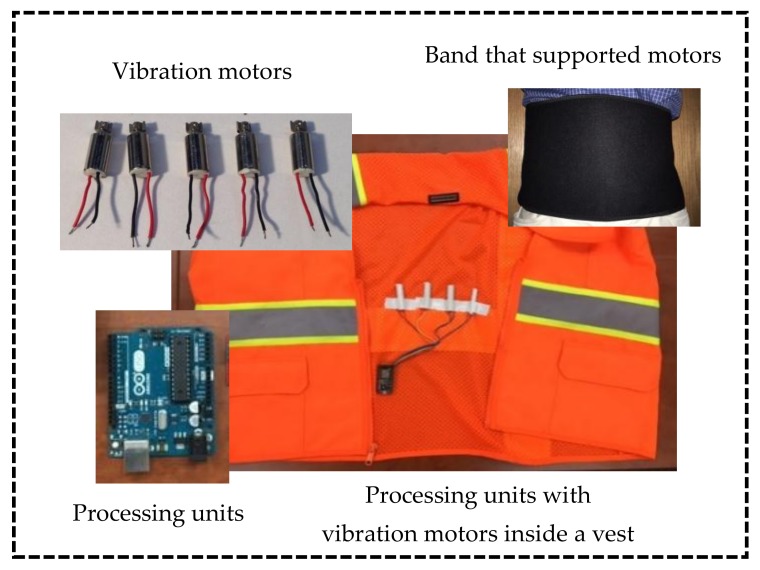
Apparatus containing vibration motors and processing units embedded inside a band, which wrapped around the waist of a test subject.

This study developed a prototype tactile communication system by using vibratory motors as the medium stimulus and communication channel as well as a control processing unit (CPU) to trigger the motors and control vibrations, which is shown in Figure 2. Several 3-V cylindrical-shaped motors were used as vibratory sensors and a Wemos D1 Wi-Fi, which is an open-source development kit, was used as a CPU. Along with the Wemos D1 Wi-Fi, an Arduino Uno Wi-Fi was tested to compare the performance after which the Wemos D1 Wi-Fi was selected for the prototype system. The CPU had a Wi-Fi feature that allowed wireless communication with other electronic devices and systems. The CPU also triggered the motors by sending various vibration signal profiles to the test subject. Since the two components (i.e., the CPU and the motors) formed the core part of the tactile-based communication system, they were able to serve the purpose of signal transmission. To deliver tactile signals to the test subject conveniently, a supporting band was used to firmly attach the motors by wrapping the band around the waist of the test subject inside a safety vest.

### 4.3. Profiles

**Figure 3:**
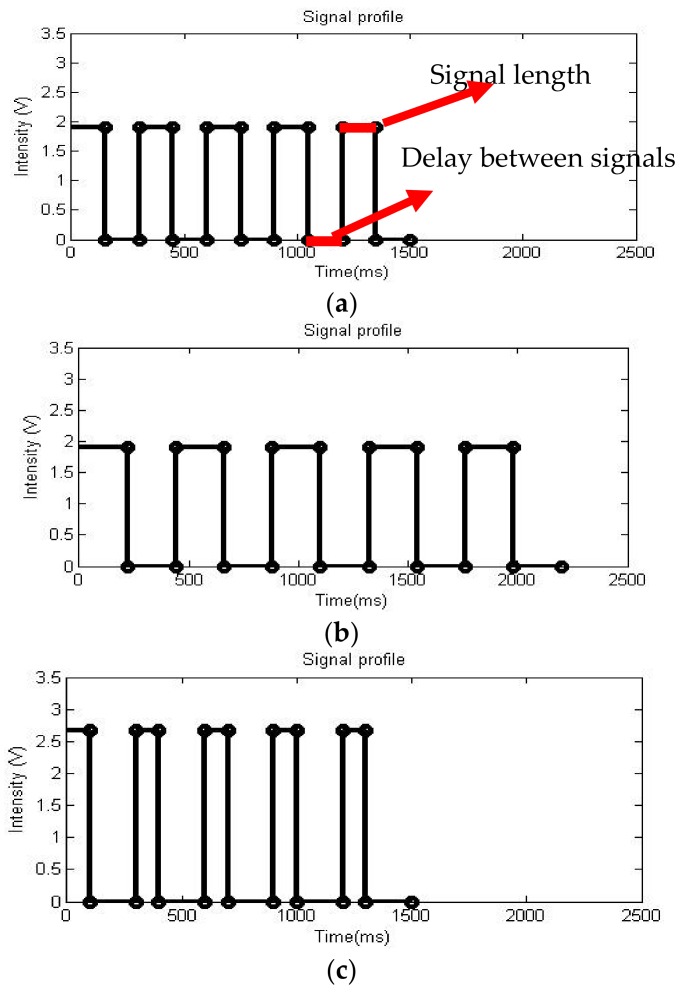
Signal variations for (**a**) Digitized Signal 1 has an intensity of 1.9 V, a signal length of 150 ms, and a delay of 150 ms. (**b**) Digitized Signal 2 has an intensity of 1.9 V, a signal length of 220 ms, and a delay of 220 ms and (**c**) Digitized Signal 3 has an intensity of 2.7 V, a signal length of 100 ms, and a delay of 200 ms.

In order to understand the perception ability of humans with regard to vibratory signals, this study explored three parameters composed of vibratory signals including intensity, signal length, and delay between consecutive pulses. Figure 3 shows three examples of simple signal profiles, which were constructed of different signal intensities, signal lengths, and time delays between the signals. Any differences in any of these parameters produced a unique signal profile. This implied that the number of possible signal generations could be close to infinity because it is technically possible to introduce many small changes to one signal profile to create many others. However, although any two signal profiles are technically different, they might not be different at the perception level of humans. This is because, by nature, people rely on their peripheral sensory organs, which have limited sensitivity. For example, one peripheral such as human eyes may not be sensitive enough to distinguish a difference of 1 mm in length between two long sticks. 

To build a strong foundation on the types of signals to be used in a tactile-based communication system, this study used a technique of signal variation to identify the perception ability of test subjects for various categorized signal profiles. In the prototype system, various signal profiles were created by manipulating the three parameters of the signal profile (see Figure 3).

The 3-V vibratory motors, which were cylindrical in shape, could cover voltage ranges from approximately 1.3 V to 3.1 V. Any triggering signals below 1.3 V were difficult to perceive since the motors did not have enough power to trigger vibration over analog signals. The amount of time for a signal communication was another critical factor because a fraction of a second could mean the difference between life and death when the safety of a construction worker was concerned. For example, if intrusion of an outside vehicle was detected at a construction work zone, workers nearby would have to evacuate within a few seconds in order to avoid collision with this vehicle. Considering this time constraint, the two parameters associated with the amount of time for signal communications, signal length, and delay between signals ranged from 75 ms to 500 ms between two consecutive pulses.

### 4.4. Signal Mapping

As discussed previously, an effort was made to establish the components of tactile language, which includes intensity, signal length, and delay between pulses in order for workers to promptly recognize tactile signals without needing a significant amount of time to perceive and process the signals. To facilitate this communication, this study adopted a signal mapping technique in which each signal component was mapped to a unique piecewise message. The aggregation of multiple signal components, which connect multiple piecewise messages, produced a complete message that conveyed the information. The key ability of the test subject was to identify and distinguish different signals so that identified signals could be mapped to their own meaning and interpreted properly. Therefore, unit tactile signals for mapping were formulated based on two principles, which are each tactile signal being easy to distinguish one from another and the duration of tactile signals being as short as possible in order to immediately react to hazards at a dynamic and vulnerable construction site.

**Figure 4:**
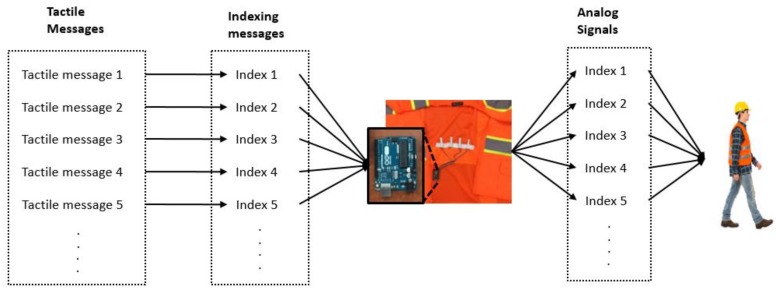
Tactile signal mapping for prompt recognition.

Figure 4 shows the conceptual overview of this signal mapping that would facilitate the tactile-based communication of workers. The mapping technique allowed us to develop indexed signals and transmission of these signals to the test subject. For example, tactile messages could include piecewise information (e.g., a hole, a truck, intrusion, or hazardous materials) and they could be indexed for identification. These indices could be identified easily by the board, which then could trigger the corresponding signal profile that represented the corresponding tactile message.

## 5. Experiment

The first experiment was conducted to take a fundamental investigation of the three signal parameters (i.e., active signal length, signal intensity, and signal delay) to determine their basic distinguishable unit signals that would be communicable in a short amount of time. To accomplish this, the research team implemented various tests to collect measurements with respect to the parameters. From the observations of this experiment and the following analysis, this work is expected to identify the digitized signal profiles and their ranges, which could be recognized by the test subjects. After identifying the basic units of the tactile signal parameters that were easily and quickly communicable, the second experiment validated whether the identified parameters could be used effectively to deliver simple information to individuals and whether those individuals could perceive the transmitted signals and take actions based on the perceived information.

### 5.1. Experiment 1: Determining the Basic Signal Units

**Figure 5:**
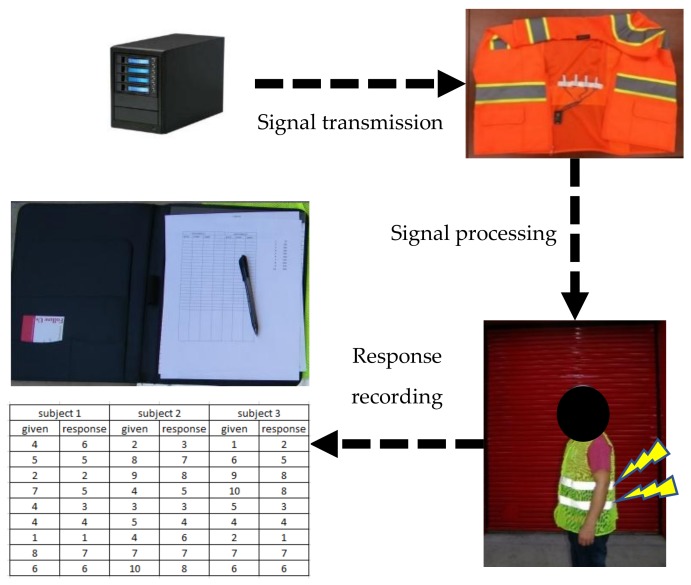
Experimental setup for Experiment 1 to determine the basic signal units.

System installation: Figure 5 shows the experimental setup. The prototype, which is an embedded sensory system, was attached to a band that was firmly tightened around the waists of the test subjects. A laptop was used to send a wireless signal to the Wemos D1 Wi-Fi board, which then simultaneously triggered a corresponding signal in the vibratory motor. A pen and a paper was given to each test subject to record indices in a number that indicated signals that they perceived through their backs.

Participants: Five test subjects were identified for their ability to sense vibratory touches on their backs. To facilitate the test, the subjects who could communicate verbally over every signal test and short-term memory of indices paired with signals were selected to participate in this study. To gain statistical significance, the tests were conducted with thousands of signal measurements through the training and testing processes. 

Signal controls: To maximize the ability of the test subjects to distinguish different signals and create an efficient tactile-based communication system, the study investigated each of the three parameters separately. The intensity was segmented into 10 equally spaced intensities (10 indices in total) from 1.3 V to 3.1 V (i.e., 1.3, 1.5, 1.9, …, 3.1). Both the signal length and the delay between consecutive pulses were initially segmented into 10 equally spaced values with each parameter holding 10 indices in total from 50 ms to 500 ms (i.e., 50 ms, 100 ms, 150 ms, …, 500 ms). See Figure 3 for examples of the signal profiles. 

Pre-testing was conducted to verify the system’s capability for signal transmission. The results indicated that the first value of 50 ms was not enough time for the subjects to perceive the triggered signals. Therefore, the first value was changed to 75 ms, which was sufficient. Table 1 summarizes signal parametric values by the order of the index. Given these segments of the parameters, signal profiles were constructed for testing. For each signal profile, the design signal pulse was relayed five times. Although these sets of signals for each of the three parameters were technically different, they might not be perceived as different by the test subjects. At the end of this experiment, these parameters were grouped into smaller clusters so that each cluster was distinct from one another.

Procedure: The first step introduced a new way of distinguishing information by using the sense of touch to the test subjects. Senses induced by the developed prototype system were not meaningful to the brain because no meaningful information had been wired to the artificially created tactile senses at this point. To bridge this gap between the senses and the perceptions of the brain, the test subjects were introduced to a training step in which an index was paired with each of the signal profiles. For each of the parameters, the test subjects went through 100 trials in incremental order for the amount of time for a signal (e.g., 50 ms, 100 ms, 150 ms, …, 500 ms). For each trial, the test subjects were informed which index was associated with the perceived tactile signal. For each subject, this training measured a total of 3000 signals. 

In the second step, the actual tests were conducted in order of intensity, signal length, and the delay between consecutive pulse tests. For each test, 6000 random numbers (1200 data x 5 subjects = 6000 data) were generated to be used as indices and paired with signal profiles. Therefore, the test subjects were asked to make 10 classifications from index 1 to 10 for 6,000 random tactile signals that they received through their backs, which is shown in Table 1. 

**Figure 6:**
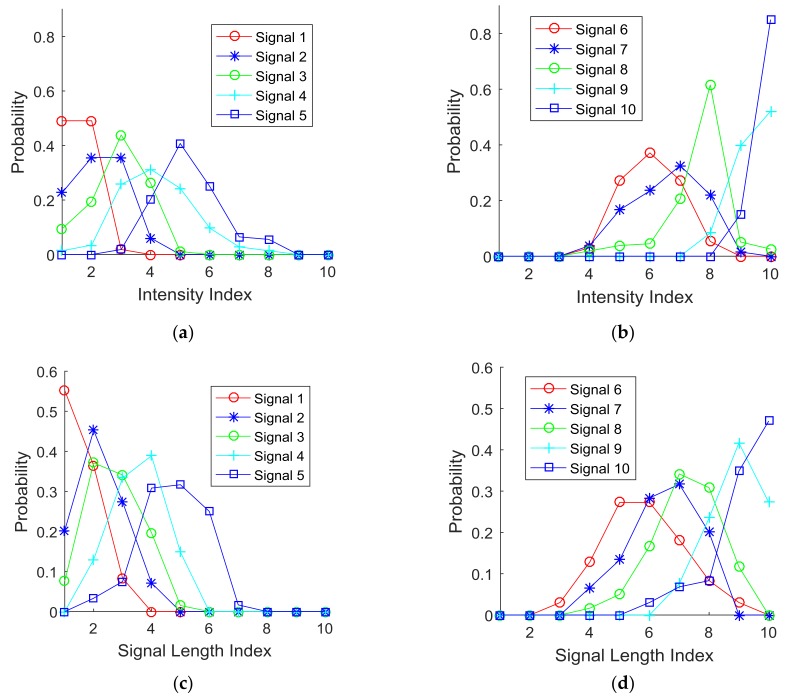

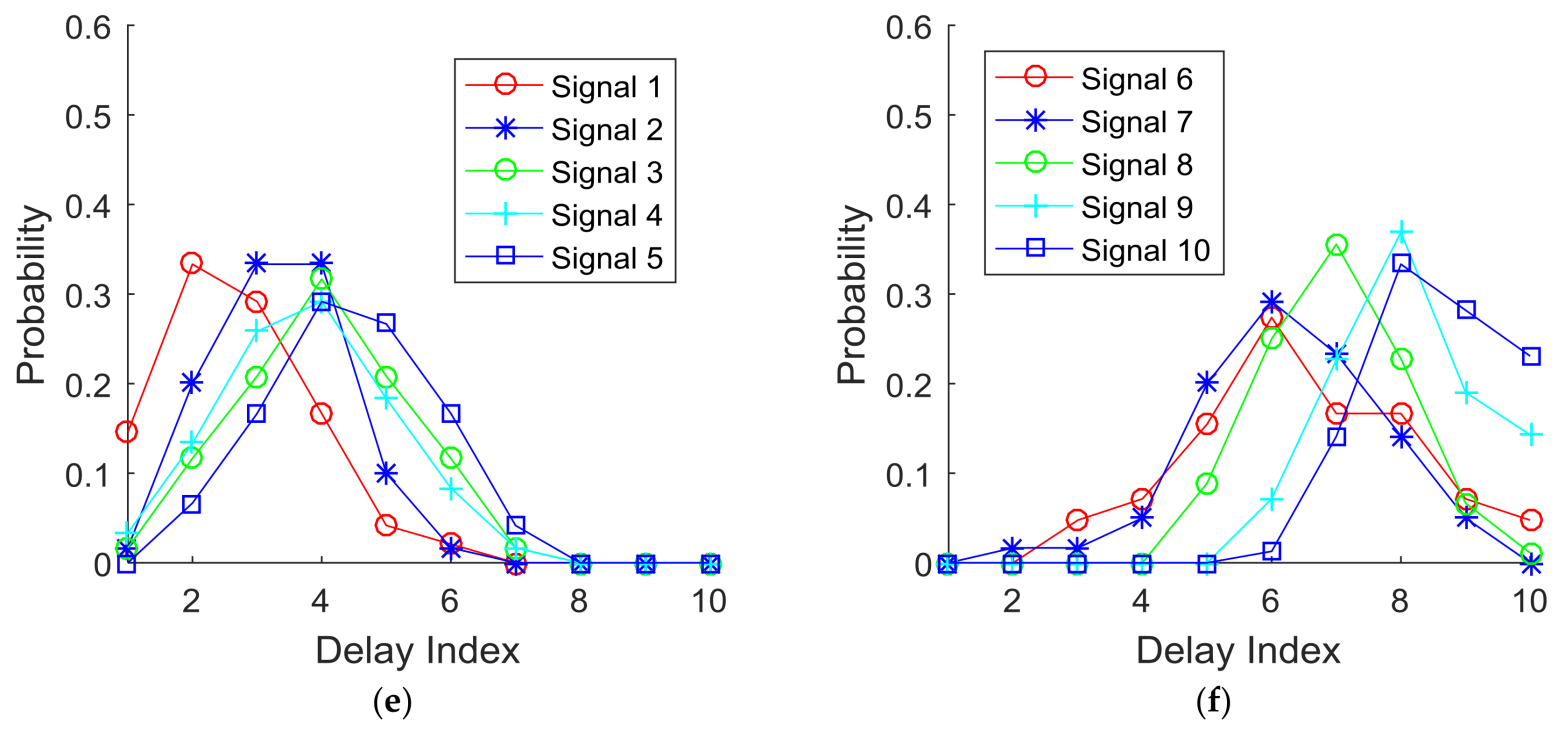
Probability density functions of the tactile parameters: (**a**) a signal intensity from 1 to 5, (**b**) a signal intensity from 6 to 10, (**c**) a signal length from 1 to 5, (**d**) a signal length from 6 to 10, (**e**) a signal delay length from 1 to 5, and (**f**) a signal delay length from 6 to 10.

Analysis: The subjects’ responses were collected and processed to construct probability density functions (PDF) for each signal index and for each signal parameter (i.e., intensity sensitivity, signal length sensitivity, and delay sensitivity). Figure 6 plots the cases of ten indices for each of the three parameters. The PDF of Signal 1 indicates the probability of the subjects’ responses to the corresponding signal index (i.e., index 1) in Table 1. Figure 6a,b plot the PDFs of signal intensity. Figure 6c,d plot the PDFs of signal length. Figure 6e,f plot the PDFs of signal delay.

**Figure 7:**
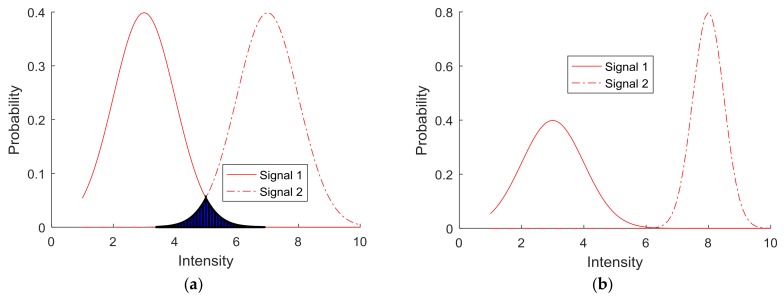
Signal distinction between (**a**) indistinguishable signals and (**b**) distinguishable signals.

The collected responses were analyzed further with respect to whether signals were distinguishable. Figure 7 illustrates this concept with two samples of probability density functions for determining distinct unit signals. Figure 7a shows two different signals. However, the subjects’ responses to the two signals overlap, which implies that there may be a potential misperception between the two signals. On the contrary, Figure 7b shows two different signals that clearly are distinguishable based on the subjects’ responses. This is an ideal situation since there are no overlapping regions. In reality, it is difficult to have such an ideal situation, which led to the development of criteria for classifying distinguishable (or perceivable) unit signals for each of the test parameters (i.e., intensity, signal length, and delay between pulses). 

**Figure 8:**
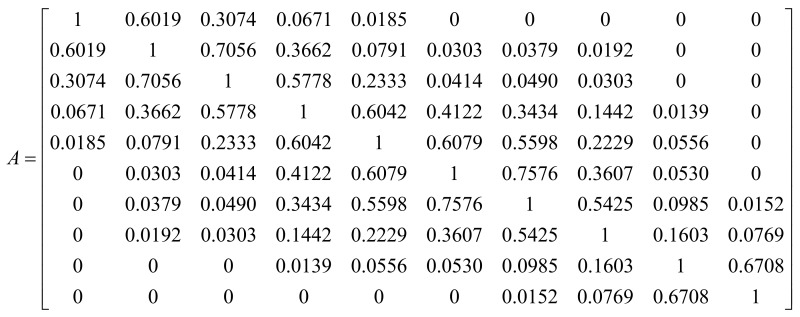
Overlapping areas between PDFs for the signal intensity parameter.

To develop criteria for identifying a distinguishable signal, which entails identifying unit signals that humans can perceive without confusion, first, the overlapping areas between all pairs of the signal indices were quantified by using the following equation.

(1) $$A_{ij}=overlapping\;area\;between\;f_{i}\left(x\right)\;and\;f_{j}\left(x\right)$$

where *f_i_*(*x*) is the probability density function of signal index *i* with respect to *x*. Figure 8 shows the results of *A_ij_* in a matrix form, A, for the signal intensity parameter. When index *i* is closer to index *j,* it is clear that the overlapping area gets larger and vice versa.

When developing the procedures for the analysis, a few sets were defined and are outlined below.

- *S* is the set containing all the signal indices. *S* = {1, 2, …, 10}
- *V_r_* is the set created from combinations of *r* elements from *S*
- *V_r_*_,*k*_ is the *k*th element in *V_r_*

As a simple illustration, *S* is set as *S* = {1, 2, 3, 4}. Then *V_r=_*_3_*—*equivalent to *V*_3_—is all the possible combinations of three elements from *S*. *V*_3_ = {{1, 2, 3} {1, 2, 4} {1, 3, 4} {2, 3, 4}}. Then *V*_3,1_ is the first element of *V*_3_, which is {1, 2, 3}. For each *V_r_*, the goal of this analysis was to find the groups of signal indices that produced minimal overlapping areas, which is computed in Equation (2) below.

(2) $$A\left(V_{r}\right)=min\left(\underset{i\in V_{r,k}}{\sum }\underset{j\in V_{r,k},i\neq j}{\sum }A_{ij}\right),\;for\;all\;k$$

Each *V_r,k_* generates one summation result of the overlapping areas defined by the PDFs associated with its signal indices. Therefore, by accounting for all *k* elements in *V_r_* shown in Equation (2), the signal indices that generate the minimal overlapping areas can be found. Since *A*(*V_r_*) is the minimized summation of the associated PDFs’ overlapping areas with respect to *r* numbers of clusters, the notation is simplified in the equation below.

(3) $$F\left(r\right)=A\left(V_{r}\right),\;for\;2\leq r\leq n=10$$

Results: As the number of clusters decreased, Equation (3) was expected to generate a smaller sum because, when the number of compared PDFs is reduced, the overlapping areas (i.e., shared probability areas between PDFs) are also reduced. Given this, in order to find the optimized number of clusters for each test parameter, the minimum summation of the shared probability areas *F*(*r*) was calculated based on Equation (3).

**Figure 9:**
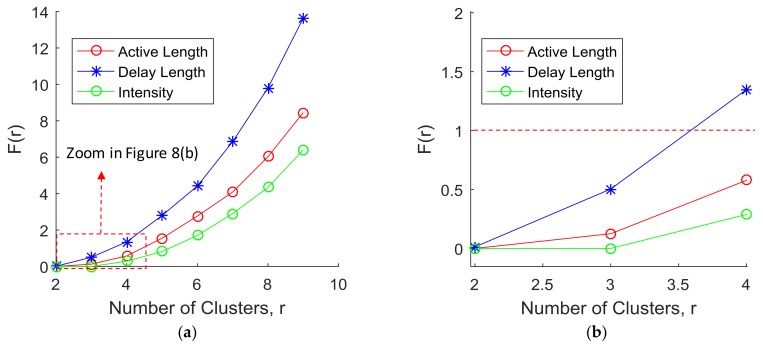
Determining the optimized (maximum) number of clusters: (**a**) number of clusters for Vs *F*(*r*) and (**b**) a close-up view of the plots below a boundary line at *F*(*r*) = 1 in Figure 9a.

The results were plotted with respect to the number of clusters in Figure 9a. When *F*(*r*) was greater than 1.0, especially with high numbers of clusters, a misperception of signals was likely to occur. The results indicate that the initial segmentation of 10 segments for each parameter was unacceptably minute since the overlapping areas were very high. Figure 9b shows a close-up view of the plots below a boundary line at *F*(*r*) = 1 in Figure 9a. This helps with understanding what happens to the signal misperception rate when the number of clusters changes. When the number of clusters, *r*, was two, *F*(*r*) of the parameters (i.e., signal active length, delay length, and intensity) was close to zero. This implies that by having only two clusters (i.e., two basic unit signals), the transmitted signals can be easily identified for the tested parameters. When the number of clusters, *r*, was three, the shared probability areas of signal active length, delay length, and intensity were 0.125, 0.504, and 0.000, respectively. While signals for the parameters including signal active length and intensity could be clearly identified with three clusters, signals for the signal delay parameter were misread approximately 50% of the time. With respect to the rate of change in misperception, this percentage rapidly increased after the point where *r* was three. Therefore, the optimized (maximum) number of clusters for the signal active length and intensity parameters was three and the optimized (maximum) number of clusters for the signal delay length was two. 

**Figure 10:**
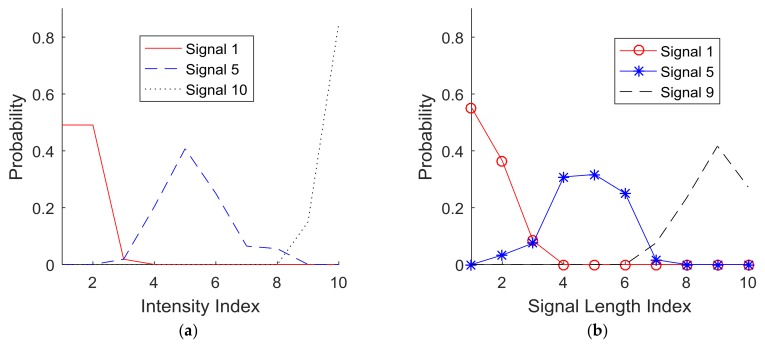
Example of clustering results when *r* = 3 for the parameters of (**a**) signal intensity and (**b**) signal active length.

**Figure 11:**
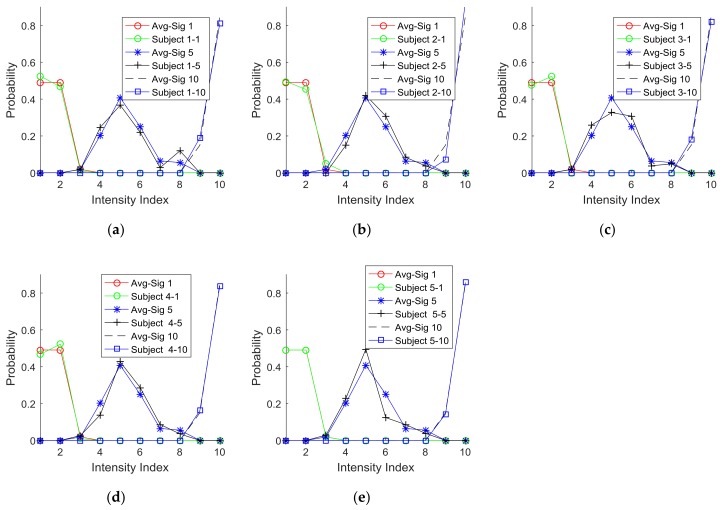
Signal intensity comparison: (**a**) Subject 1, (**b**) Subject 2, (**c**) Subject 3, (**d**) Subject 4, and (**e**) Subject 5 (Note: Avg-Sig #: average response at signal index #; Subject #-#: Subject #(first)’s response at signal index #(second)).

**Figure 12:**
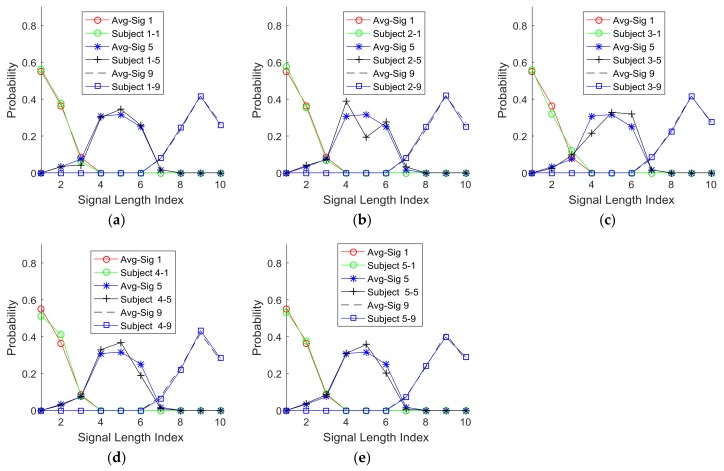
Signal length comparison: (**a**) Subject 1, (**b**) Subject 2, (**c**) Subject 3, (**d**) Subject 4, and (**e**) Subject 5 (Note: Avg-Sig #: average response at signal index #; Subject #-#: Subject #(first)’s response at signal index #(second)).

Figure 10a,b present the optimum cases (*r* = 3) of clustering the probability functions for the parameters, signal active length, and intensity. These plots show that such clusters identified by Equations (1), (2), and (3) have very small overlapping areas, which indicate high exclusivity. To further confirm the validity of this generic clustering with three classifications (*r* = 3), the individual subjects’ PDFs are plotted with respect to signal active length (indices 1, 5, and 10) in Figure 11 and intensity (indices 1, 5, and 9) in Figure 12. The comparison of these plots with the corresponding optimal cases (*r* = 3) of clustering the probability functions suggest that the optimized clusters are also well matched with each individual case. Therefore, the optimized signal units identified by clustering could serve as the basic signal units for the purpose of communication.

### 5.2. Experiment 2 for Validating Communicability of the Identified Basic Unit Signals

Experiment 1 was conducted to identify optimized signal clusters that could serve as basic unit signals for tactile communication in a short amount of time. As follow-up validation, Experiment 2 was implemented to determine whether the identified parameters could be used effectively to deliver simple information to individuals and whether individuals can perceive transmitted signals and take actions requested by using this perceived information. 

System installation and participants: The system setup was the same as for Experiment 1. Three of the five subjects participated in this experiment.

Procedures: Since this test was conducted to investigate the effectiveness of the identified basic unit signals of the tactile signal parameters, the signals with specific messages were mapped and used to deliver specific information to the test subjects. Although eight basic unit signals were identified from Experiment 1, six unit signals were used with three from the intensity parameter (Test 1) and three from the active signal length parameter (Test 2). These signals were mapped to specific meanings, which is shown in Table 2. Because of the relative simplicity in the parameter for signal delay length, this parameter was not included in the validation. 

**Figure 13:**
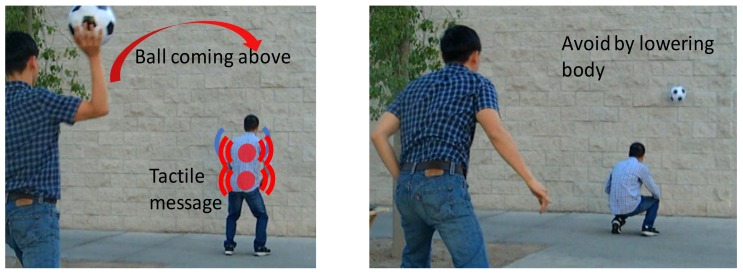
Validation of the second experiment: test of the effectiveness of the identified parameters for delivering quick messages for hazard avoidance.

The research group emulated a specific hazard condition that could be represented by cases such as dropping heavy tools and for vehicle intrusions. This hazard condition was simulated by throwing a ball (to represent dropped tools or intruding vehicles) to the test subjects, which is shown in Figure 13. As soon as the ball was thrown, tactile signals indicating the situation were transferred wirelessly to the subjects and the subjects took a required action based on the perceived tactile signals. Each of the three tested subjects repeated each test 50 times. For example, the ball was thrown randomly to one of three directions with respect to each test subject 50 times for each test and the test data were recorded for assessment. Each test collected 150 data sets and the total data collected was 300 data sets.

Result and discussion: Table 3 and Table 4 summarize the results of this experimental validation. In the tests, the failure rates were counted when the test subjects could not avoid the thrown ball properly. Individual assessment on the tests show three and seven failures for Test 1 and Test 2, respectively. In other words, the overall accuracies were 98% and 95.3% for the two tests. Out of 300 trials, 10 failures occurred for two types of cases, which are explained below.

(1) The signal was properly perceived but the subject did not react properly (8 times) and
(2) The signals were sent with a delay (2 times).

These specific failures were reasonable. The first case with eight failures reflected possible human mistakes, which could be improved with training. The second case had only two failures out of 300 for less than a 1% failure rate. This does not disqualify the benefit of the tactile communication system, which is to provide an additional sensing ability to construction workers. All the failure rates were lower than 6% and the overall success rate of the two test cases was 96.7%. The failure rate based on the signal intensity test (Test 1) was relatively lower than that based on the signal active length test (Test 2). This implies that the signal intensity parameter performed better as a tactile medium when used exclusively.

Given the special working conditions of construction workers under which they often fail to recognize impending hazards due to their limited sensing abilities, the validation results provided evidence that hazard sensing abilities can be improved with sensory aids. The identified signal parameters derived from the first experiment demonstrated their ability to facilitate simple communication, which was validated in the second experiment. 

## 6. Conclusions

Since the environmental conditions of construction sites are dangerous, workers are always exposed to construction hazards. Although many researchers have developed sensing technologies to identify safety issues, workers are unable to perceive information about impending hazards in the appropriate amount of time. One of the challenges that workers have in recognizing impending hazards is their limited sensing abilities. Vision and hearing are the main senses workers use to perceive their surroundings and recognize alerts about hazards. However, these two senses are easily interfered with at construction sites due to their dynamic, loud, and complicated environments. When workers are engaged at a construction site, their sensing abilities to hazards are limited.

This study explored a new method for construction workers to sense hazards by using tactile signals and their associated parameters. This idea was derived from neuroscientific evidence that a human’s brain can extend its perception range through sensing training. This study explored this in a slightly different way from past neurological studies to adopt a tactile-based sensing approach for construction workers. The research team developed an embedded tactile sensory system on a Wemos D1 Wi-Fi board connected to vibrating motors. Tactile signals were investigated for three parameters, which include signal active length, delay, and intensity. In particular, a range from 50 ms to 500 ms was selected for the parameters of signal active length and delay length due to time-sensitive safety events in construction. Signal intensity was also controlled by a defined voltage range varying from 1.3 V to 3.1 V. 

The first experiment identified distinguishable sets of signal parameters by data collection, processing, and analysis. Five subjects were first tested to obtain large sets of data and the clustering method was formulated to find the optimal number of unit signals for message mapping. As a result, three, three, and two distinguishable signals were identified for the signal intensity, active signal length, and delay parameters. The second experiment simulated a construction hazard by throwing a ball at three test subjects who were asked to react based on perceived tactile signals when the ball was thrown. The failure rates for the cases with two parameters, which included signal intensity and signal active length, were lower than 6% (i.e., higher than 94%). This means that the proposed system could offer the potential to expand the workers’ sensing ability for hazard recognition and improve their chances of avoiding impending hazards. One of the most significant findings of this work was identifying and validating basic tactile unit signals for use in construction safety in a timely manner. 

The second experiment showed that the system communication was reliable since the delay rate was less than 1%. The mode of the system between the motors and the controlling server was a paired mode, which contributed to a reliable wireless connection. Therefore, the number of slaves may be limited by the capacity of the server. In addition, while the identified unit signals and their effectiveness in short communications were essential, this study has not completely constructed a full level of a vibratory-based language that conveys more meaningful information. Therefore, further extensive research would be needed in order to develop full message delivery protocols, training, and learning, which would require multiple rounds of experiments. This might lead to widespread application in specific areas regarding construction safety. In particular, regarding vocabulary and lexicon, more unit tactile signals (or more words) should be explored and validated. Such exploration and validation on more complex signal combinations may enable more complete levels of communication for construction workers. However, this communication may entail high error rates at an initial state because responses for each subject may deviate more when compared with the responses from simplified signals. Therefore, it will require the design of sophisticated signal combinations to generate disguisable messages through vibrations to reduce the gap between different individual responses. In addition, time sensitivity and learning rates with respect to training should be explored further.

## Figures and Tables

**Table 1:** Three parameter values of the signal profiles.

Index	Signal Intensity (V)	Signal Length (ms)	Signal Delay (ms)
1	1.3	75	75
2	1.5	100	100
3	1.7	150	150
4	1.9	200	200
5	2.1	250	250
6	2.3	300	300
7	2.5	350	350
8	2.7	400	400
9	2.9	450	450
10	3.1	500	500

**Table 2:** Simple tactile signal mapping for hazardous situations.

Test 1		Test 2	
Signal Index	Mapped Meaning	Signal Index	Mapped-Meaning
Intensity 1	“ball thrown left so move right”	Active Length 1	“ball thrown left so move right”
Intensity 5	“ball thrown right so move left”	Active Length 5	“ball thrown right so move left”
Intensity 9	“ball thrown above so sit down”	Active Length 10	“ball thrown above so sit down”

**Table 3:** Test 1 results on the effectiveness of the identified signals for quick message delivery.

Test 1 (Intensity)				
Required Action	Failure Count			
	Subject 1	Subject 2	Subject 3	Total
“Move Right” (index 1)	1 (out of 18)	0 (out of 17)	1 (out of 17)	2 (out 52)
“Move Left” (index 5)	0 (out of 15)	0 (out of 17)	1 (out of 18)	1 (out 50)
“Sit Down” (index 9)	0 (out of 17)	0 (out of 16)	0 (out of 15)	0 (out 48)
Correct rate	98%	100%	96%	98%

**Table 4:** Test 2 results on the effectiveness of the identified signals for quick message delivery.

Test 2 (Active Signal Length)				
Required Action	Failure Count			
	Subject 1	Subject 2	Subject 3	Total
“Move Right” (index 1)	1 (out of 17)	1 (out of 16)	0 (out of 16)	2 (out 49)
“Move Left” (index 5)	1 (out of 16)	1 (out of 18)	1 (out of 18)	3 (out 52)
“Sit Down” (index 10)	0 (out of 17)	1 (out of 16)	1 (out of 16)	2 (out 49)
Correct rate	96%	94%	96%	95.3%
